# An exploratory study of dynamic foot shape measurements with 4D scanning system

**DOI:** 10.1038/s41598-023-35822-0

**Published:** 2023-05-27

**Authors:** Li-ying Zhang, Kit-lun Yick, Mei-jun Yue, Joanne Yip, Sun-pui Ng

**Affiliations:** 1 School of Fashion and Textiles, The Hong Kong Polytechnic University, Hung Hom, Kowloon, Hong Kong China; 2 Laboratory for Artificial Intelligence in Design, Hong Kong Science Park, New Territories, Hong Kong, China; 3 School of Professional Education and Executive Development, The Hong Kong Polytechnic University, Hung Hom, Kowloon, Hong Kong China

**Keywords:** Computational biology and bioinformatics, Health care, Engineering, Mathematics and computing

## Abstract

Accurate and reliable foot measurements at different stances offer comprehensive geometrical information on foot, thus enabling a more comfortable insole/footwear for practical use and daily activities. However, there lacks investigations on continuous deformation of foot shape during the roll-over process. This study analyses the foot deformation of 19 female diabetic patients during half weight bearing standing and self-selected walking speed by using a novel 4D foot scanning system. The scanning system has good repeatability and accuracy in both static and dynamic scanning situations. Point cloud registration for scanned image reorientation and algorithms to automatically extract foot measurements is developed. During the foot roll-over process, maximum deformation of length and girth dimensions are found at first toe contact. Width dimensions have maximum deformation at heel take off. The findings provide a new understanding of foot shape changes in dynamic situations, thus providing an optimal solution for foot comfort, function and protection.

## Introduction

Accurate foot measurements are important to enable footwear and insole fit and comfort. The foot shape in the half weight bearing (HWB) condition provides an important reference source for customised insole designs^1,2^, while the subtalar joint in the neutral position is widely used for foot orthoses in clinical casting^3^. Undoubtedly, the foot shape changes with different loads in standing and walking conditions^4–6^. It is widely accepted the footwear should match with the foot shape for optimal fit and wear comfort^7^. However, the lack of dynamic foot information has compelled designers to consider dynamic foot deformation information based on their own assumptions and experience when designing shoe lasts^8^. Based on self-reported through interview and surveys, foot ulcers are frequently caused by new shoes, friction caused by footwear materials, and inappropriate fit^9^.

Nevertheless, little research has been conducted on the changes in foot shape with motion^10–16^ due to the high cost of dynamic scanning systems. The dynamic foot scanning system proposed in Coudert et al.^15^ consists of six cameras that work in a synchronised manner. However, socks must be worn or else the surface of the feet must be painted with ink to solve the problem of lack of texture, which is undesirable in a clinical setting. A technique for taking the cross-sectional (ball, instep, and heel) measurements of the foot during walking was proposed in Kimura et al.^17^. Multi-view stereo matching was used to capture the dorsal surface of the foot. The scanning system was then upgraded so that a camera and an LCD projector could be used to reduce noise and simplify the detection process of the corresponding points^13^. Vidmar et al.^12^ also developed a 4D foot scanning prototype by using commodity hardware and RGB­-D cameras to make the 4D scanning more accessible, with the use of active stereoscopic vision technology. A novel structured-light prototype system was proposed in Thabet et al.^14^, in which a video sequence of the foot during a single step is inputted, and resulted in the output of a 3D reconstructed plantar of the foot that corresponds with the inputted information. However, these systems either have a low sample rate of 14–15 Hz only^12,16^ and deliver poor image quality due to limited number of cameras, or can only scan the geometry of the dorsal or plantar surface^14,15^. This study analyses the deformation of the diabetic foot during motion by using a 4D measurement method (3dMD LLC, Atlanta, GA) to capture dense surface images in static conditions and dynamic shapes during motion with sparse landmarks. The method can capture 360-degree full foot images at a sampling rate of 40 Hz.

In addition to scanning technology, the post-processing of scanned images has a major influence on the image quality and further analysis of the data related to the feet. However, due to the problems with 3D scanning such as scanning angle (cannot measure surface that is beyond the line of sight of the scanner), and differences between object size and scanning distance^18^, the traditional manual registration and measurement process is time-consuming and prone to errors. The use of different scanning systems has however resulted in a large volume of point clouds, so algorithms have been developed for point cloud registration to increase the data processing efficiency and accuracy in previous studies^18–22^. Point cloud registration has been widely used in many computer vision tasks, like 3D reconstruction^23,24^, object detection^25^, pose and motion estimation^26–28^, autonomous driving^29,30^, robotic manipulation^31^, medical imaging^32–34^, etc. It is an important step to merge multiple data sets into a globally unified model, or map an unknown data set onto a known data set to identify its features or estimate its pose.

Besl and Mckay^20^ described a well-known method for point cloud registration based on the iterative closest point (ICP) algorithm. This algorithm repeatedly selects the corresponding pairs of points between the target and source point clouds, then calculates the optimal translation, rotation, and scaling up or down, and finds the transformation parameters between the corresponding pairs of points by using the least squares method. This process is repeated until there is an optimal match between the two sets of point clouds to meet various metric criteria, like the root-mean-square error (RSME)^35^. Another robust probabilistic method for point cloud registration based on a coherent point drift (CPD) algorithm was introduced by Myronenko and Song^22,36^. The CPD algorithm is used to consider the alignment of two point sets as a probability density estimation problem^22,37^, where the probability value of 1/0 represents the true/false correspondence respectively, and a larger probability refers to greater certainty of the correspondence. Given that there are two point sets **M** and **S**, they represent the distribution of **M** as a Gaussian Mixture Model (GMM), where the corresponding likelihood function peaks when **M** is fully aligned with **S**. In this study, the CPD algorithm is used for registering foot images to a standard coordinate for measurement purposes, and then the key foot measurements related to the shoe/insole design are extracted automatically.

Therefore, the primary aims of this study are:

1. To validate the repeatability and accuracy of the 3dMD foot scanning system,
2. To explore a suitable approach for foot image reorientation based on point cloud registration so that key foot measurements can be automatically extracted, and
3. To analyse foot roll-over shapes for patients with diabetes.

## Methodology

### Dynamic foot scanning system

**Figure 1:**
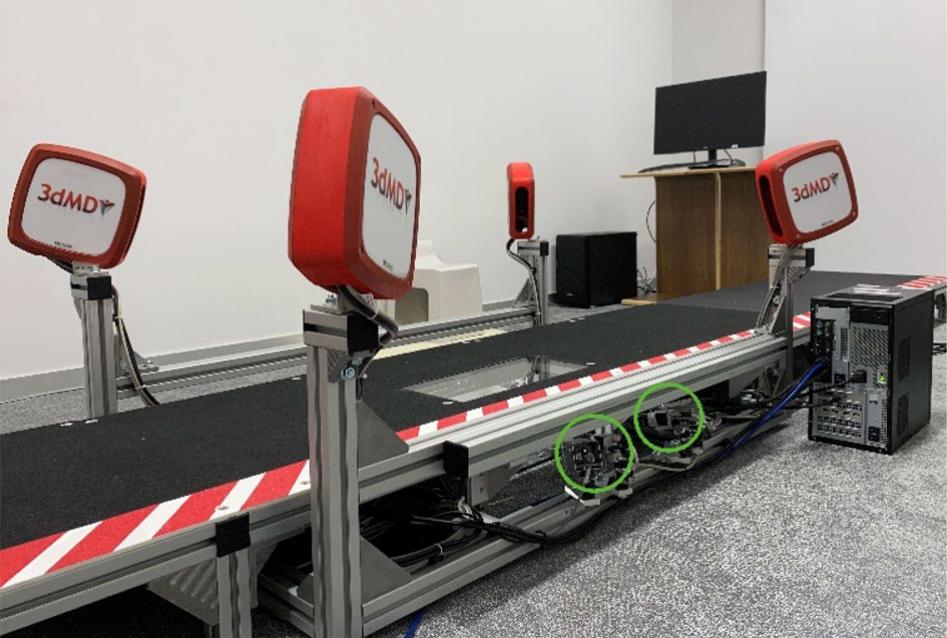
3dMD foot scanning system.

The 3dMD foot scanning system (3dMD LLC, AG, USA) is also known as a 4D foot scanning system, which has the additional dimension of time as opposed to the three dimensions of 3D scanning systems. The system contains 10 machine vision cameras, including dedicated cameras (green circle labelled in Fig. 1) that capture the plantar of the foot with projection through glass or customised speckle patterns. The speckle patterns enable the accurate reconstruction of the foot shape when in contact with the glass (length * width: 42 * 19 cm). They provide a 360-degree geometric shape of the entire foot for both the surface of the dorsal and plantar of the foot with articulation and movement during locomotion, and a progressive sequence of 3D foot images (40 fps) over a period of time can be recorded, which enables the operator to select the optimal frame in real time for immediate analysis or render the entire image sequence for further evaluation and use. The system rendering engine incorporates information from all camera viewpoints per frame so there is no manual stitching required. The accuracy of the 3D foot images from this system can reach 0.7 mm or higher. A 100% LED lighting environment also ensures the safety and comfort of the subjects during longer scanning sessions.

### System validation

Accuracy and repeatability are two important indicators of the robustness of measurements in measuring systems. According to ISO 5725-1:1994, accuracy is the degree of agreement between the test result and the true or accepted reference value. Precision is defined as the degree of agreement between test results obtained under the same stipulated conditions^38^. Good repeatability can ensure as few errors as possible that are caused by measuring systems randomly. Therefore, a reliable measurement system should have both good repeatability and the ability to provide accurate measurements.

**Figure 2:**
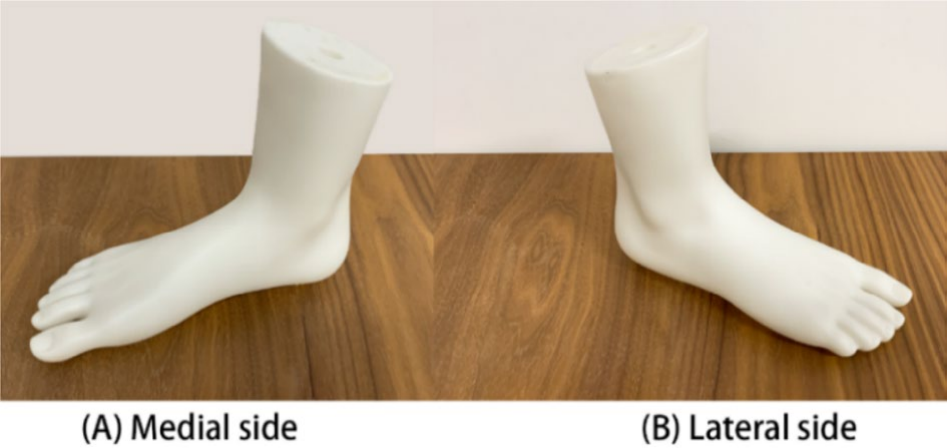
Foot model.

In this study, the repeatability of the 3dMD foot scanning system is examined through repeated scanning of the same object under the same conditions. The accuracy is examined by comparing the difference in the measurements of the same object between the system and a market-available scanner with a relatively high degree of accuracy. To prevent unavoidable subjective errors caused by soft tissue deformation and body sway, a rigid foot model (see Fig. 2) and a rigid ball (ping pong ball) with a circumference of 40 mm are used to assess the measurement performance of the system in static and dynamic situations respectively.

#### Repeatability test

**Figure 3:**
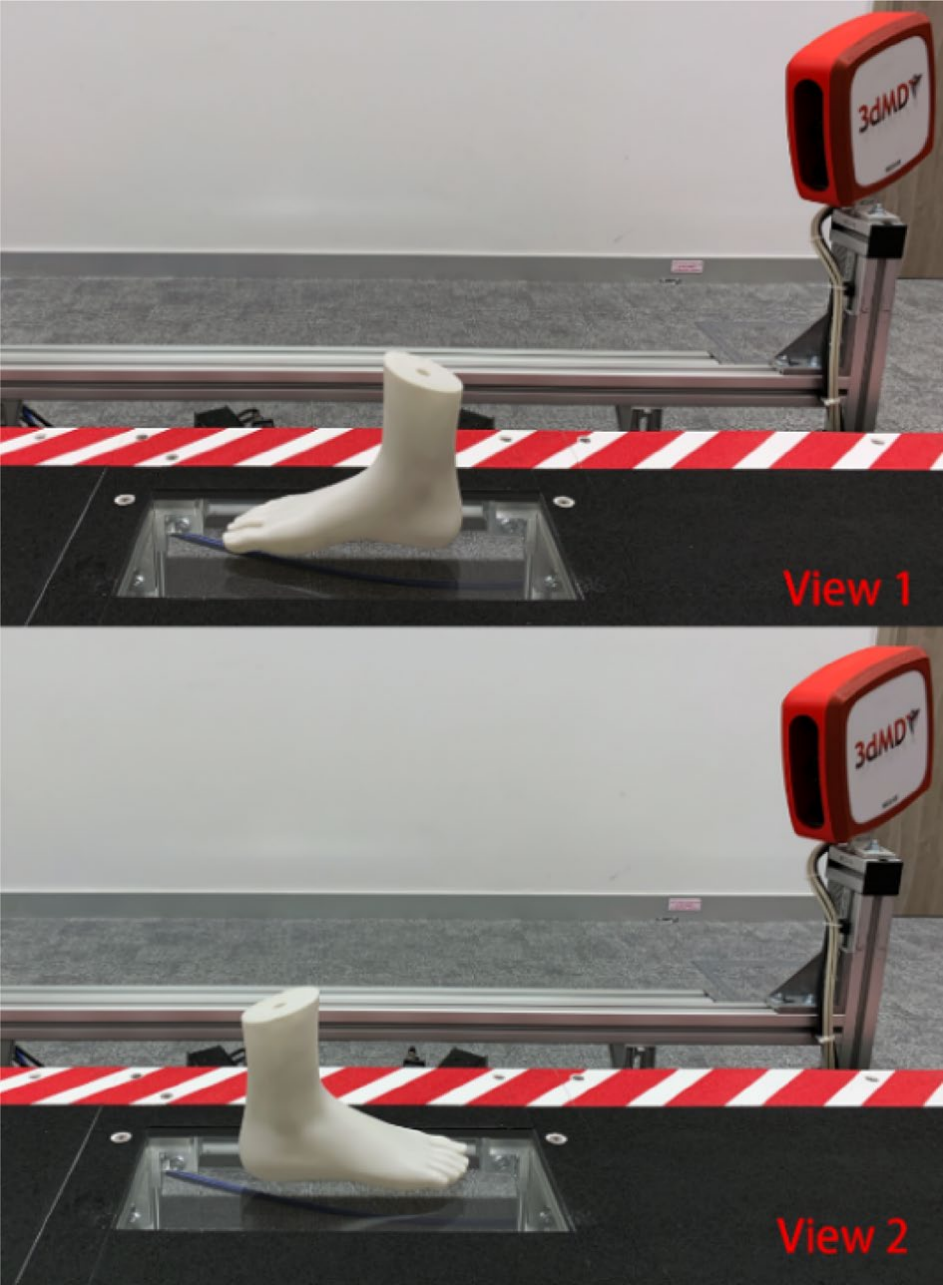
Two different scanning views.

Repeatability is defined as the level of consistency between measurements of the same object taken under the same scanning conditions by using the system. The foot model and ball were therefore scanned three times by the same operator under the same scanning conditions to assess the repeatability of the system. The foot model was scanned with a self-weight bearing at 2 different views (the angle of the two views is 180°) respectively, see Fig. 3. The rigid ball was also scanned when rolling through the platform from two different directions (the angle of the two directions is 180°). The rolling speed is 1.4 m/s^39^, which is close to the average walking pace of humans. The system was switched off and on between each scan.

The raw scanned data of the foot model and ball were processed by using Geomagic Design X software to create meshes. These meshes were randomly selected and superimposed to calculate the mesh deviations of various pairs for repeatability assessments: (1) among the meshes scanned in the same view/direction, and (2) among the meshes scanned in a different view/direction. The accumulated areas (in % of the total mesh area) of the mesh deviations between each pair were calculated at 0.1 mm increments^40,41^. The mesh deviation is the difference between the scanned images created by the system.

#### Accuracy test

**Figure 4:**
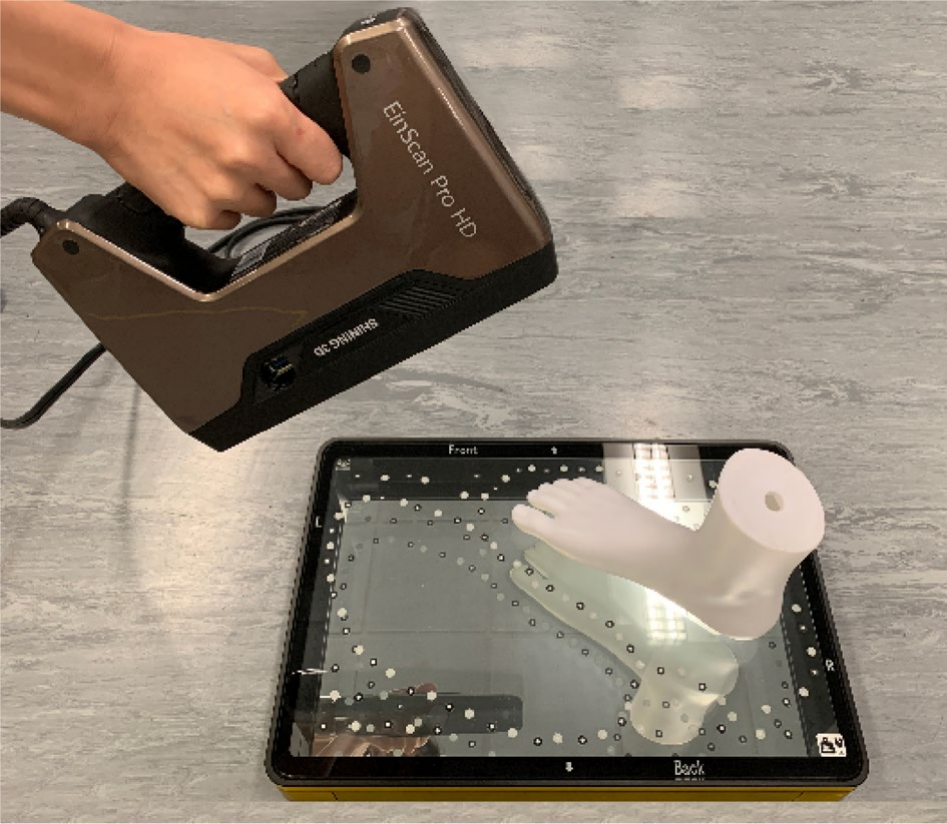
EinScan Pro handheld 3D scanner with foot station.

Generally, accuracy is defined as the proximity of a measurement to its true or tolerant value. In this study, the 4D foot scanning system is compared with the EinScan Pro handheld 3D scanner (SHINING 3D®) with a foot station^1^, see Fig. 4. This scanner can process up to 3,000,000 points per second under the handheld scan mode. The handheld scanner shows a similar resolution and accuracy as the fixed scanner by using a minimum point distance setting of 0.2 mm. The accuracy of the scanned foot data with the use of this scanner is up to ± 0.5 mm. Both surface of the dorsal and plantar of the foot can be scanned simultaneously by using the foot station.

A rigid foot model was scanned three times by the same operator who is well trained to use both the handheld scanner and 4D foot scanning system. Scanned images from both scanners were randomly selected and compared by using Geomagic, and the accumulated areas (in % of the total mesh area) of the mesh deviations were calculated at 0.1 mm increments between the selected pair. The mesh deviation is the measurement error between scans created by the two scanners. In addition, the circumference of scans of the ball from the 4D foot scanning system was measured 5 times to obtain the mean value for comparison with the original circumference of the ball so as to validate the accuracy of the system in a dynamic condition.

### Foot scanning and measurements in standing and walking conditions

#### Experiment

**Figure 5:**
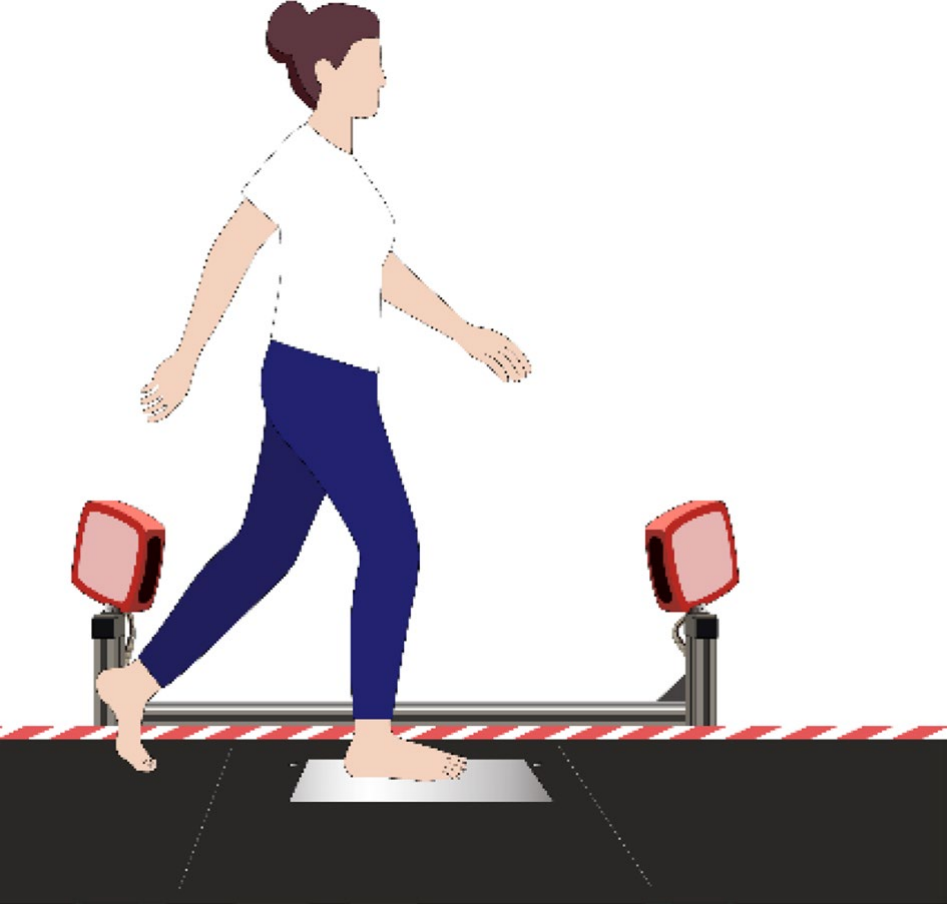
Dynamic foot scanning.

A total of 19 female subjects between 57 and 75 years old (mean: 66, SD: 5) participated in the study. The height, weight and BMI (body mass index) of the involved subjects are 152.0–166.0 cm (mean: 157.6, SD: 4.3), 42.0–72.0 kg (mean: 55.2, SD: 7.3) and 18.2–30.8 kg/m^2^ (mean: 22.3, SD: 3.2) respectively. The dominant foot, which is determined to be the foot that kicks a ball habitually^42^, was scanned 3 times during stance with HWB and self-selected walking speed conditions respectively by using the 4D foot scanning system, see Fig. 5. The subjects have Type 1 or 2 diabetes mellitus (DM) in the early stages (self-reported with a diagnosis from a clinical physician) and the inclusion criteria^43,44^ are those with no history of ulcers or neurological disorders (except neuropathy), and able to walk a length of 20 m repeatedly without a walking aid. Subjects who show the presence of active foot ulcers were excluded^45^. This study was reviewed and approved by the Human Subjects Ethics Sub-committee of The Hong Kong Polytechnic University (Reference Number: HSEARS20200128001). All methods were performed in accordance with the relevant guidelines and regulations. Information on the experimental requirements were provided to all of the participants and written informed consent was obtained from all the participants before the experiment commenced.

#### Foot anthropometric measurements

**Figure 6:**
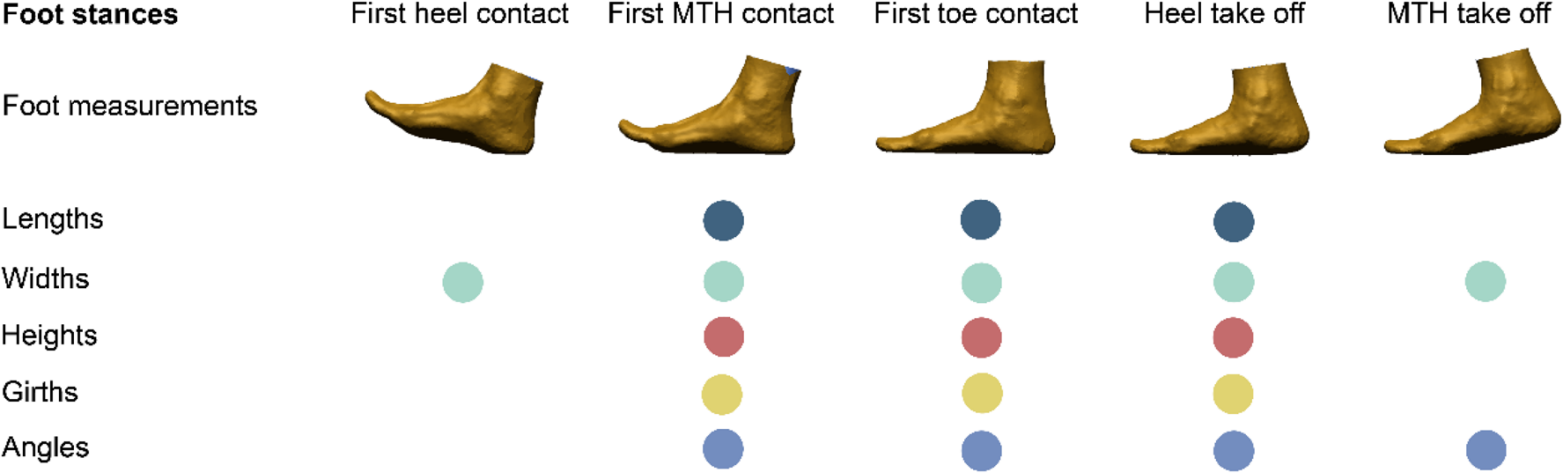
Foot anthropometric measurements for 5 frames during ground contact.

Thirteen key foot anthropometric measurements were extracted for an in-depth analysis of the foot deformation, including length: foot length (FL), medial ball length (MBL), lateral ball length (LBL); width: anatomical ball width (ABW), orthogonal ball width (OBW), and orthogonal heel width (OHW); height: ball height (BH), instep height (IH); girth: anatomical ball girth (ABG), and instep girth (IG); and angle: ball angle (BA), toe1 angle (T1A), and toe5 angle (T5A)^5,6^. According to the foot roll-over pattern defined by Blanc et al.^46^ and Barisch-Fritz et al.^5^, five representative frames at first heel, MTH contact, and toe contact, and heel and MTH take off respectively were selected from the plantar perspective of the complete roll-over process, and their corresponding foot measurements, see Fig. 6.

### Point cloud registration and automatic foot measurements

Instead of processing the scanned images and taking foot measurements manually by using Geomagic, new approaches of point cloud reorientation for 3D foot image registration and automated measurement are proposed, to improve the efficiency of foot image analysis and measurement.

#### Point cloud filtering

Raw data obtained from 3D scanners is often corrupted with noise and outliers^47^. These errors can reduce the accuracy of the point cloud representation and post-processing, thus causing issues in determining the local point cloud features (e.g., normal vectors or curvature rate of change at sampled points), which can lead to incorrect values and then errors in point cloud alignment, data measurement, and other post-processing tasks. Therefore, the first step of scanned image processing is 3D point cloud filtering, which is used to remove the noise and outliers^48,49^. A large number of variant filters have been proposed for different types of surfaces and noise models^48,49^, including the Statistical Outlier Removal filter^50,51^, Bilateral filter^52,53^, Radius Outlier Removal^54^, and Voxel Grid filter^55^, etc. However, none of these methods are appropriate as they require an iterative trial-and-error process to choose the algorithm and the parameters based on the scanned surface and noise characteristics.

**Figure 7:**
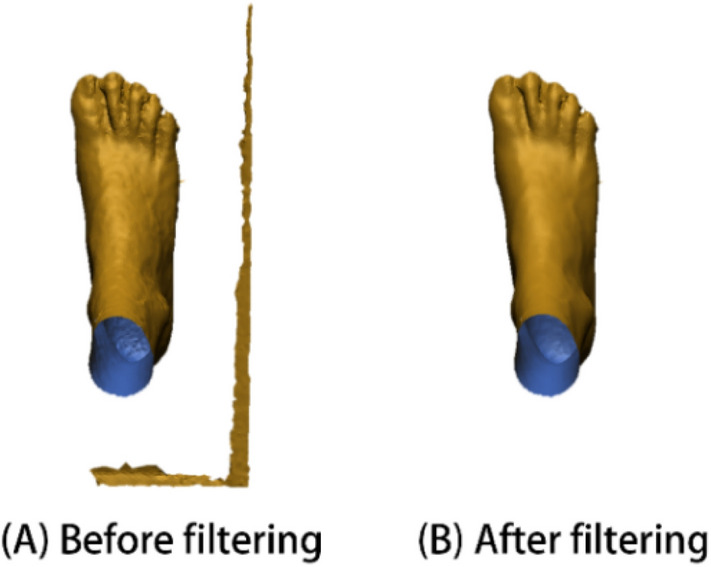
Example of data comparison (**A**) before and (**B**) after filtering.

In this study, the noise and outliers of the foot images are removed by using Statistical Outlier Removal (Sor) filter before registration. This is carried out by calculating the neighbours of each point. The sparse outliers that do not meet the criteria are trimmed. For each point p_i_ (i = 1,…, n) in the input dataset, the mean distance *d*_*i*_ from p_i_ to all its neighbours are calculated, see Eqs. (1) and (2). The result is assumed to be a Gaussian distribution with mean and standard deviations. The points that are not within a σ standard deviation from the mean distance are defined as outliers and removed from the dataset^48^, which means if *d*_*i*_ > *d*_*mean*_ + 1*σ, then *p*_*i*_ is removed. Figure 7 shows an example of data comparison before and after filtering.

1 $${d}_{mean}=\frac{\sum_{k=1}^{n}{d}_{k}}{n}$$

2 $$\sigma = \frac{1}{n-1}\sqrt{{\sum }_{k=1}^{n}{\left({d}_{i}-{d}_{mean}\right)}^{2}}$$

#### Point cloud registration

**Figure 8:**
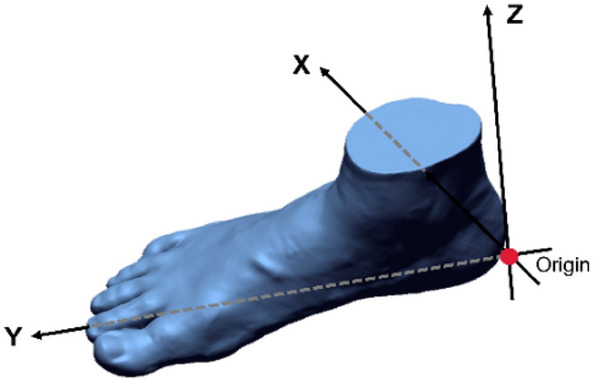
Standard position of the 3D foot image: (1) x-y plane: the plantar of the foot contact plane and, (2) the y-axis: x-y plane projection line of the foot axis (the line that joins the pternion and second metatarsal-phalangeal joint and, (3) origin: the projection point of pternion on the x-y plane.

After filtering, the 3D image should be re-aligned to a standard position based on local system to take the foot measurements^40,56^, see Fig. 8. First, the coordinate of the point set—first toe contact frame, was manually aligned to the standard position as the target point set. Then the other point sets were aligned to the target point set respectively by finding the points that correspond to each other. In this study, the alignment between the target and other point sets is regarded as a density estimation problem based on the CPD method.

The target and other point sets are the two 3D point sets in this study: **M**_J×3_ = (*m*_*1*_*, m*_*2*_*, …, m*_*J*_)^T^ and **S**_K×3_ = (*s*_*1*_*, s*_*2*_*, …, s*_*K*_)^T^, where J and K are the number of points in the two point sets respectively. The points in the target point set **M** were generated by using the GMM. The other point set **S** as the GMM centroids need to fit **M** by maximising the probability of GMM centroids **S** to generate **M**. This is to force **S** (GMM centroids) to move coherently as a group to preserve the topological structure of the point set^22^.

**Figure 9:**
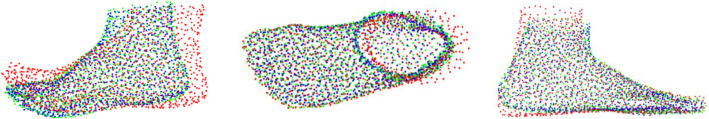
Example of 3D image registration (red denotes **S**, green denotes **M**, blue denotes registration result).

In this study, good approximation to the distance function between **S** and **M** is developed based on the CPD algorithm^57^. The problem of point set registration is regarded as distance function optimization, which is to minimize the errors to find the best space transformation between the point sets. An example of a 3D image registration result is shown in Fig. 9.

#### Automated extraction of foot measurement

**Figure 10:**
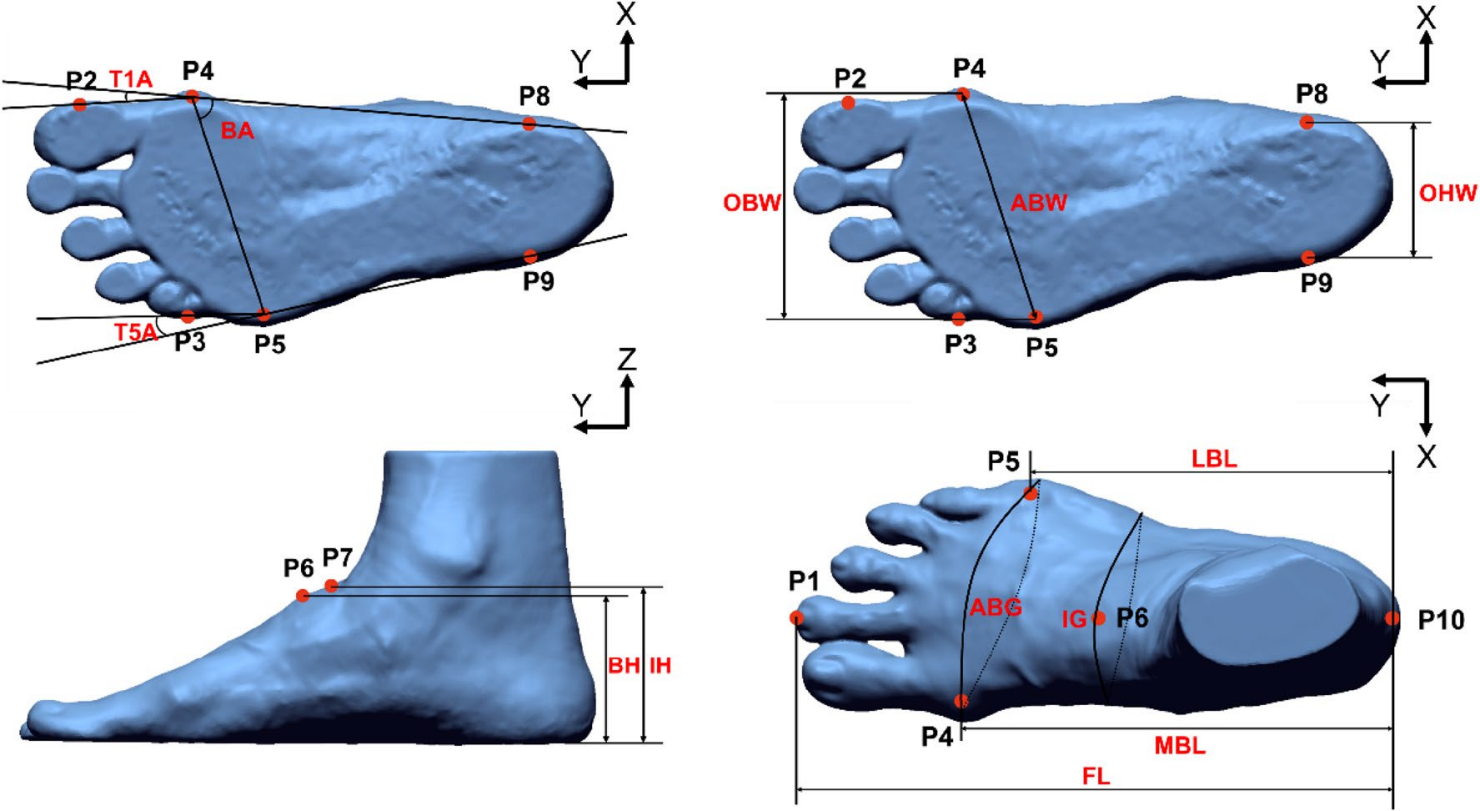
Thirteen foot anthropometric measurements.

Using image registration, all five selected frames were reoriented to a standard coordinate. According to the definition of each foot measurement^6^, the key anatomical points (P1–P10) were first extracted from each frame automatically. A total of thirteen foot measurements were taken, by using Eqs. (3) - (13) (Fig. 10). Also, the automated foot measurements based on 5 subjects were compared with manual measurements to examine the consistency of the two methods.

**Figure 11:**
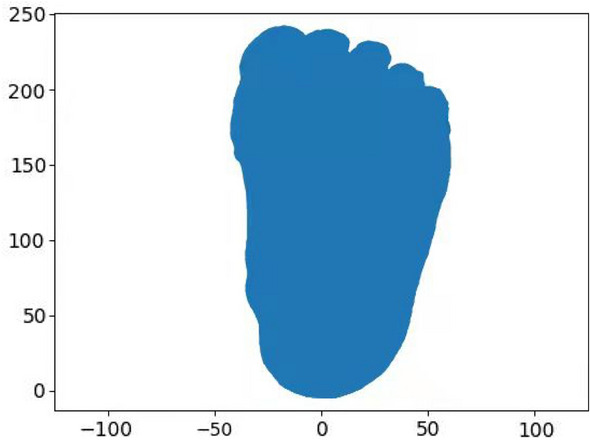
Projection outline of foot in X-Y plane.

The key points (P1-P10) extracted from the foot images include: P1 and P10 which are the maximum and minimum Y values along the Y-axis. The foot outline projection in the X-Y plane (Fig. 11) consists of bumps extracted by using the Graham algorithm^58^. P2 is the point with the maximum absolute X value when the Y value are between P1 and P4. P3 is the point with the maximum absolute X value when the Y value is between P1 and P5.

P4 and P5 are the points with the maximum and minimum X values along the X-axis.

P6 and P7 are the points with the maximum Z values along the Z-axis when the Y values are 0.5 and 0.618 in the FL respectively.

P8 and P9 are the points with the maximum and minimum X values along the X-axis with a 14–20% FL.

The FL, MBL and LBL are the distance between the pternion P10 and P1, P4, P5 respectively along the X-axis.

3 $$FL=\left|{Y}_{P1}- {Y}_{P10}\right|$$

4 $$MBL=\left|{Y}_{P4}- {Y}_{P10}\right|$$

5 $$LBL=\left|{Y}_{P5}- {Y}_{P10}\right|$$

The ABW is the distance between P4 and P5; OBW is the distance between P4 and P5 projected onto the X-axis and OHW is the distance between P8 and P9 projected onto the X-axis.

6 $$ABW=\sqrt{{({Y}_{P4}- {Y}_{P5})}^{2}+{({X}_{P4}- {X}_{P5})}^{2}}$$

7 $$OBW=\left|{X}_{P4}- {X}_{P3}\right|$$

8 $$OHW=\left|{X}_{P9}- {X}_{P8}\right|$$

The BH and IH are the distance of P6 (61.8% of FL) and P7 (50% of FL) to the X-Y plane along the Z-axis, respectively.

9 $$BH=\left|{Z}_{P6}\right|$$

10 $$IH=\left|{Z}_{P7}\right|$$

The BA is the angle between lines $P4P5$ and $P4P8$; T1A is the angle between $P2P4$ and $P4P8$; and T5A is the angle between $P3P5$ and $P5P9$.

11 $$BA=\mathrm{arccos}\left(\frac{{\overline{P4P5}}^{2}+{\overline{P4P8}}^{2}-{\overline{P5P8}}^{2}}{2\overline{P4P5}*\overline{P4P8}}\right)$$

12 $$T1A=180^\circ -\mathrm{arccos}\left(\frac{{\overline{P2P4}}^{2}+{\overline{P4P8}}^{2}-{\overline{P2P8}}^{2}}{2\overline{P2P4}*\overline{P4P8}}\right)$$

13 $$T5A=180^\circ - \mathrm{arccos}\left(\frac{{\overline{P3P5}}^{2}+{\overline{P5P9}}^{2}-{\overline{P3P9}}^{2}}{2\overline{P3P5}*\overline{P5P9}}\right)$$

The ABG is the circumference that passes through P4 and P5 along the Z-axis, and the IG is the circumference that passes through P6 along the Z-axis. As shown in Eq. (14), Pi is the point at the cross sections (ABG, IG), and *N* is the number of points.

14 $$girth= \sum_{i=0}^{N}\overline{PiPi+1} , i=\mathrm{0,1}\dots N$$

### Data analysis

All of the foot measurements were analysed by using SPSS Statistics 21 software (IBM Corp., Armonk, NY, USA). A Shapiro-Wilk test was used to examine the normality of the 13 foot measurements. The results showed that all of the foot measurements are normally distributed (*P* > 0.05) in the different conditions. Intraclass correlation coefficients (ICCs) were used to determine the consistency of the automatic and manual measurement methods. A paired sample *t*-test was used to determine the significance between foot measurements during walking and in the stance condition with HWB. The significance level of the statistical analysis was set at 0.05.

## Results

### Repeatability of 3dMD foot scanning system

To evaluate the repeatability of the system, the 3D images of the foot model and ball were paired and selected randomly from 2 different scanning views/directions. Two randomly selected images were aligned using global and automatic alignment methods successively in Geomagic. Details of the pairs and results of the mesh deviations are listed in Table 1.

**Table 1:** Randomly selected pairs and mesh deviations for repeatability assessment.

Selected pair	Percentage of overlapping area (Mesh deviation within 0.5 mm)	Percentage of overlapping area (Mesh deviation within 1 mm)
Static scanning (foot model)		
Pairs of View 1	98.83%	99.84%
Pairs of View 2	98.86%	99.80%
Pairs of Views 1 & 2	96.08%	99.56%
Dynamic scanning (ball)		
Pairs of Direction 1	98.94%	99.95%
Pairs of Direction 2	98.46%	99.95%
Pairs of Directions 1 & 2	98.92%	100%

For the static scanning of the foot model, 98.83% of the areas of overlap between randomly selected images from View 1 has a deviation less than ± 0.5 mm, and 99.84% within ± 1 mm. Pairs in View 2 showed similar results; the overlapping areas between the meshes is 98.86% with a deviation less than ± 0.5 mm, and 99.80% within ± 1 mm. The overlapping areas of images randomly selected from Views 1 and 2 are up to 96.08% with a deviation less than ± 0.5 mm, and 99.56% within ± 1 mm.

For the dynamic scanning of the ball, 98.94% of the overlapping area between randomly selected images from Direction 1 has a mesh deviation less than ± 0.5 mm, and 99.95% within ± 1 mm. Pairs from Direction 2 showed similar results. The overlapping areas between the meshes is 98.46% with a deviation less than ± 0.5 mm, and 99.95% within ± 1 mm. The overlapping areas of images randomly selected from 2 different directions up to 98.92% with a deviation less than ± 0.5 mm, and 100% within ± 1 mm.

### Accuracy of 3dMD foot scanning system

**Figure 12:**
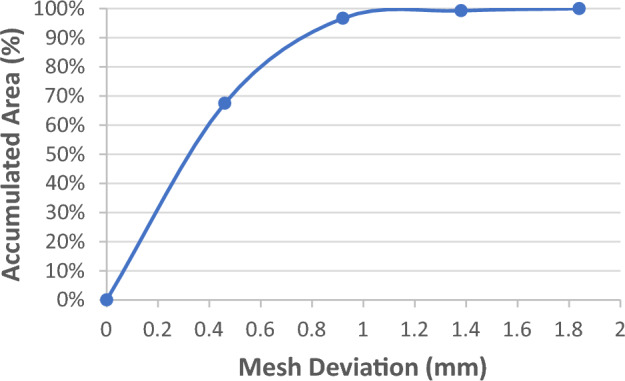
Mesh of foot model comparison between 4D foot scanning system and handheld scanner.

Meshes of the foot model from the 4D foot scanning system and EinScan Pro handheld 3D scanner were compared to examine the accuracy of the system under a static situation. It was found that 96.66% of the mesh overlapped between images randomly selected from the 2 scanners with a deviation less than ± 1 mm, see Fig. 12. For the accuracy validation of dynamic scanning, the circumference of ball scans randomly selected from 2 directions were measured 5 times by using Geomagic. The mean value of their measured circumference is over 99%, quite close to the original value of 40 mm.

### Comparison of foot measurements between automatic and manual methods

In this study, a total of 13 foot measurements of 5 subjects have been measured by using automatic and manual measurement methods respectively. ICC analysis was used to check the consistency of the foot measurements measured with the automatic and manual methods. In Table 2, the ICC value of each foot measurement is higher than 0.8 (*P* < 0.001), which indicates that the automated foot measurement shows a good agreement with the manual measurement.

**Table 2:** Intraclass correlation coefficient of foot measurements obtained from automatic and manual measurement methods.

Foot measurement	Intraclass correlation^b^	95% Confidence interval		F test with true value 0			
		Lower bound	Upper bound	Value	df1	df2	Sig
Foot length	.856^a^	.058	.971	32.212	9	9	.000
Medial ball length	.874^a^	.002	.977	1043.185	14	14	.000
Lateral ball length	.892^a^	− .001	.981	655.763	14	14	.000
Anatomical ball width	.836^a^	.004	.968	2704.874	19	19	.000
Orthogonal ball width	.801^a^	− .003	.959	494.694	19	19	.000
Orthogonal heel width	.855^a^	.003	.972	1456.289	19	19	.000
Instep height	.896^a^	− .004	.981	491.653	14	14	.000
Ball height	.896^a^	− .013	.981	259.255	14	14	.000
Ball angle	.932^a^	.007	.988	1162.003	14	14	.000
Toe 1 angle	.851^a^	− .009	.972	319.943	14	14	.000
Toe 5 angle	.917^a^	− .010	.985	282.972	14	14	.000
Anatomical ball girth	.939^a^	.014	.990	7636.438	14	14	.000
Instep girth	.931^a^	.012	.988	6516.468	14	14	.000

### Foot measurements of diabetic patients

#### Participant information

The description statistics of the 19 female diabetic participants are listed in the Table 3, including age, weight, height, body mass index (BMI), foot size and years of diagnosis.

**Table 3:** Description statistics of participants (n = 19).

Variable	Mean	Standard deviation	Maximum	Minimum
Age (years old)	66	5	75	57
Weight (kg)	55.2	7.3	72.0	42.0
Height (cm)	157.6	4.3	166.0	152.0
BMI (kg/m^2^)	22.3	3.2	30.8	18.2
Foot size (EUR)	38	1	41	37
Years of diagnosis (DM)	13	1	31	10

#### Comparison of foot measurements: dynamic vs. static conditions

The maximum (MaxDyn) and minimum (MinDyn) values of each foot measurement during dynamic walking were extracted to analyse the changes of each foot measurement during whole ground contact. In addition, the MinDyn and MaxDyn of the foot measurements were compared with those of HWB standing through a paired sample *t*-test respectively, see Table 4.

**Table 4:** Paired sample *t*-test of foot measurements: static versus dynamic conditions.

Foot measurement (unit: mm)	MaxDyn		HWB standing		MinDyn		MaxDyn versus MinDyn (%)	MaxDyn versus HWB (%)	MinDyn versus HWB (%)
	Mean	SD	Mean	SD	Mean	SD			
Foot length (FL)	239.4	7.4	236.0	8.1	236.1	7.4	**1.4**	**1.4**	0.1
Medial ball length (MBL)	177.0	5.2	175.4	4.4	173.5	5.0	**2.0**	**1.0**	− 1.0
Lateral ball length (LBL)	152.3	4.0	150.0	3.5	150.6	3.7	**1.2**	**1.5**	0.4
Anatomical ball width (ABW)	99.6	4.9	95.8	5.7	91.1	4.6	**9.3**	**3.9**	**− 5.0**
Orthogonal ball width (OBW)	93.4	4.7	92.9	4.4	89.0	4.5	**4.9**	0.5	**− 4.2**
Orthogonal heel width (OHW)	52.5	4.5	50.1	4.7	50.5	4.5	**4.1**	**4.8**	0.7
Instep height (IH)	62.7	4.6	60.5	3.0	58.5	4.6	**7.2**	**3.7**	− 3.2
Ball height (BH)	49.5	5.4	47.1	3.0	47.6	5.5	**4.1**	**5.2**	1.0
Ball angle (BA)	78.4	9.7	75.7	9.8	69.9	9.7	**12.1**	3.5	− 7.6
Toe 1 angle (T1A)	17.6	5.8	13.0	4.5	13.7	5.5	**28.4**	**35.7**	5.7
Toe 5 angle (T5A)	13.7	3.6	10.2	4.3	10.8	0.6	**26.6**	**33.3**	5.3
Anatomical ball girth (ABG)	226.5	13.4	218.2	14.1	222.7	13.4	**1.7**	**3.8**	2.1
Instep girth (IG)	238.8	13.3	228.1	13.9	232.6	13.2	**2.7**	**4.7**	2.0

^a^Groups with significant differences at the 0.05 level are bolded.

**Figure 13:**
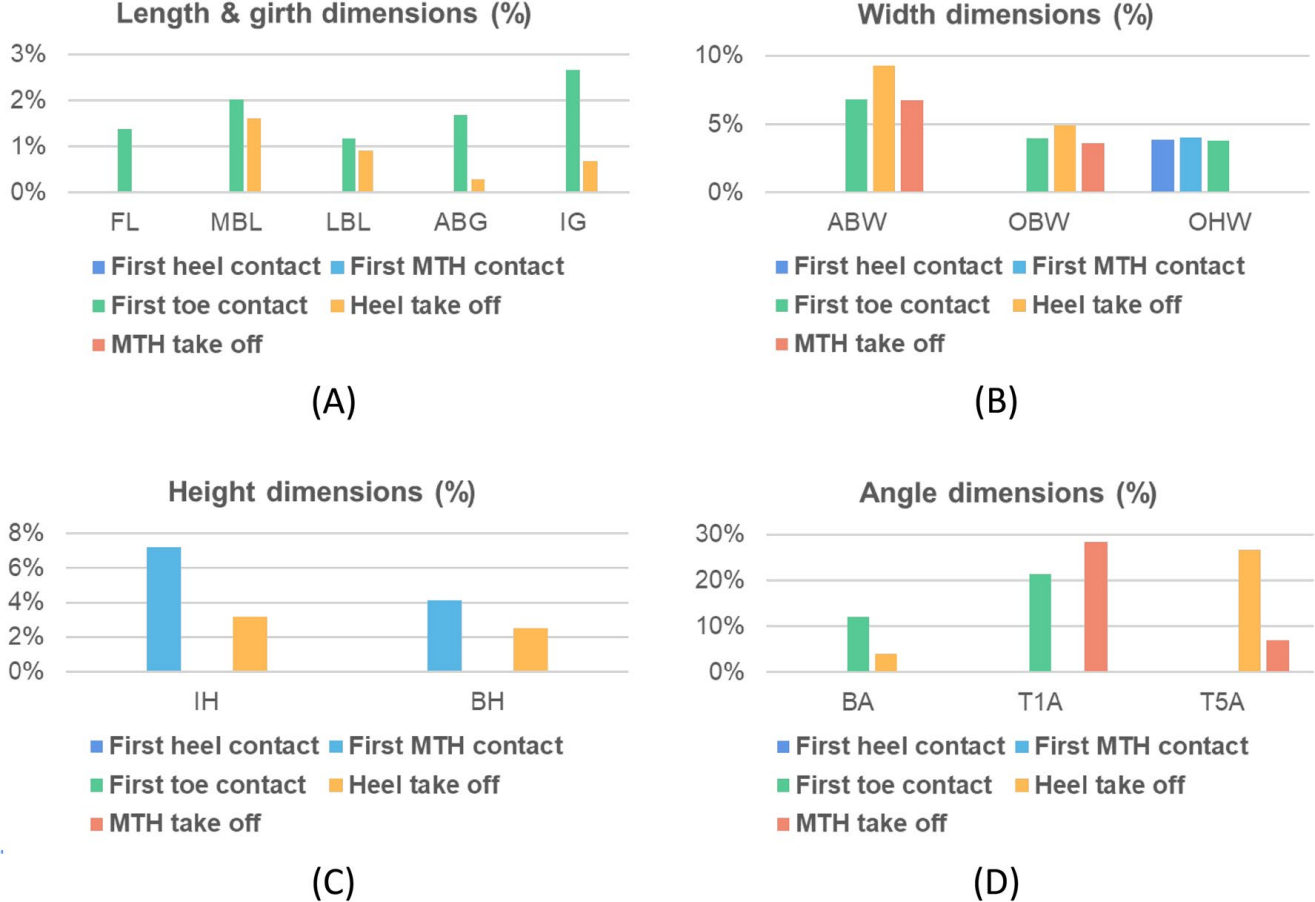
Change percentage of foot measurements during roll-over process: (**A**) length & girth dimensions; (**B**) width dimensions; (**C**) height dimensions; and (**D**) angle dimensions.

As shown in Table 4, when the MaxDyn and MinDyn are compared, the measured foot values significantly increase from 1.2 to 28.4% in dynamic situations. From the first heel contact to MTH take off, the foot dimension changes are continuously measured and compared with MinDyn. Major increases in foot length and girth are found at the stance of first toe contact, while its dimension change in foot height is minimal. The highest increment in foot width is found at heel take off stance, see Fig. 13.

Then the MaxDyn and MinDyn are also compared with HWB standing respectively. The measured foot values significantly increase 0.5–35.7% from HWB to MaxDyn with the exception of orthogonal ball width and ball angle. While no significant differences are found with each foot measurement between MinDyn and HWB, except for ball width.

## Discussion

### Scanning performance of 3dMD foot scanning system

As shown in Table 1, the repeatability of randomly selected images from the same scanned views is similar (98% within ± 0.5 mm) in both static and dynamic scanning situations, which indicates that this system shows high repeatability from the same scanning views. The overlapping area from different static scanning views is slightly less than that from the same view, which might be attributed to the fact that View 2 is specialized for left foot scanning with the current equipment setting. The foot model is not a homogeneous object and therefore the repeatability of the scanned images in the two different views is slightly lower compared to that of the scanned images from the same view. For the homogeneous ball, the repeatability of images from 2 different directions does not have this problem. The repeatability of the 4D foot scanning system excels that of 4D foot reconstruction system (4DFRS), which is a dynamic foot reconstruction system developed by Thabet et al.^59^, and has proven reconstruction differences that average 2.44 mm and 2.81 mm in static and dynamic situations respectively.

In addition, Fig. 12 indicates that the accuracy of the system is similar as that of the handheld scanner with static scanning. However, meshes overlap less within a stricter deviation, but still meet the foot measurement tolerance in footwear manufacturing^60^. For dynamic scanning, the measurement of scans is well close to the original value. The accuracy of this system is higher than the dynamic foot scanning system proposed in Novak et al.^61^, the accuracy of which can achieve a scanning height and width better than 1.12 mm, scanning girth better than 1.73 mm, and scanning angle better than 2.41°.

### Foot deformation at different stances while walking

In this study, compared to the foot shape at static situation, continuous changes of each foot measurement were investigated and found to have significant deformations at dynamic situation. For deformation of lengths (FL, MBL, and LBL): The dynamic lengths are larger compared to static stance with HWB. As shown in Table 4, the extension degree of MBL is larger than LBL. Toe allowance is crucial for shoes to enable a natural roll-over process^62^. Footwear manufacturer used to leave enough space in front of the toes for wearing comfort following the fashion trends based on subjective judgement. From this study, a tolerance of 3 mm in the lengths should be considered as dynamic allowance to optimise the fit of the toe box during walking.

For deformation of widths (ABW, OBW, and OHW): Compared to static stance with HWB, the widths reach the maximum value at heel take off stance. From this study, a tolerance of 5 mm in ball width should be considered for toe box shape design to accommodate the foot deformation. The heel width increased 4.8% during walking compared to static stance with HWB. Heel cup is recommended to enclose the heel soft tissue by providing additional confinement for the heel^63^.

For deformation of height dimensions (IH, BH): The arches collapse with increased loads upon the foot during walking^64,65^. Arch pads and metatarsal pads, which accommodate the morphological plantar deformation during locomotion can ensure the plantar fully contact the insole for better distribution of plantar pressure. As shown in Table 4, on the basis of arch pad thickness at HWB standing, a tolerance of 3-5 mm is further recommended for motion comfort.

For deformation of angles (BA, T1A, T5A): Besides toe allowance, the ball angle is also an important measure to determine the flex line location for shoes. In this study, it is interesting to find that the ball angle tends to be obtuse during walking. The angle between the flex and medial lines of the shoe for diabetics is thereby suggested to be approximately 90°. The forefoot seems more pointed since the toe angles increase during walking, especially at final push stances. The deformation of TA1 is slightly higher than TA5, which can be attributed to the fact that Hallux Valgus is commonly found in the feet of diabetic patients^66^. However, for shoe/insole design, pointed forefoot contour is not recommended due to toes spread out during standing.

For deformation of girths (ABG, IG): The dynamic girth is larger than static stance with HWB, which might result from the contractions of intrinsic and extrinsic foot muscles^67^, as well as plantar fascia and ligaments deformation during walking^68^. As shown in Table 4, a 10 mm allowance should be considered to optimise the shoe girth fit during walking.

In this study, the continuous changes of foot shape were analysed from 5 representative stances, to investigate the dynamic allowance in design of diabetic footwear with optimal fit and comfort during walking. Generally, the findings from this study remedy the gap in the analysis of static foot deformation. Nevertheless, there are still some limitations in this study. First, the 5 frames which represent the whole ground contact are selected through visual observation, so manual errors are unavoidable even though this task is done by the same person. All of the frames of the foot roll-over process rather than a few of them are measured automatically for analysis. Therefore, the error caused by manual selection can be avoidable. Secondly, the number of diabetic subjects involved in this study is small (only female subjects), so the results cannot be generalized. More subjects can be recruited to analyse dynamic foot deformation in future studies.

## Conclusion

This study has introduced an advanced 4D foot scanning system with good repeatability and accuracy for foot measurements in both standing and walking contexts. In comparison to traditional 3D scanning, the system can comprehensively and efficiently compare and visualise the changes in foot shape at different regions and during different stances of walking. In this study, the foot geometry changes and deformation of the feet of diabetic patients during their self-selected walking speed are continuously measured and compared by using the advanced scanning system. Compared with the foot measurements taken at static foot stance, foot length and girth dimensions at first toe contact and foot width at heel take off provide good references of foot shape changes during walking in design of diabetic footwear. Conventional footwear designs based on foot shapes taken with static stance in no/half weight bearing conditions inherently result in fit problems during walking, thus adversely affecting wear comfort and increasing the risk of foot pain and pressure. This study contributes by providing a practical recommendation for diabetic footwear and insole designs. Apart from adding toe allowance in designing the toe box and flex line, arch and metatarsal pads as well as a heel cup can be taken into consideration to reduce the risk for diabetic foot ulcers caused by excessive plantar pressure and deformation at dynamic situations. Skin-marker based motion capture is a frequently used method for foot movement analysis at different dynamic situations^69^. However, it is time-consuming and costly and detailed deformation of soft tissue cannot be captured^28^. Extended studies will be conducted to extract the information of foot motion functions from the new marker-less 4D scanner to give detailed recommendation on therapeutic insole design for diabetes.

## Data Availability

Data are available upon reasonable request. If you want to request the data from this study, please contact corresponding author, Dr. Kit-lun Yick.
